# Method developments to extract proteins from oil palm chromoplast for proteomic analysis

**DOI:** 10.1186/s40064-015-1576-4

**Published:** 2015-12-22

**Authors:** Benjamin Yii Chung Lau, Santanu Deb-Choudhury, James D. Morton, Stefan Clerens, Jolon M. Dyer, Umi Salamah Ramli

**Affiliations:** Advanced Biotechnology and Breeding Centre, Malaysian Palm Oil Board, No. 6, Persiaran Institusi, Bandar Baru Bangi, 43000 Kajang, Selangor Malaysia; AgResearch Lincoln Research Centre, Christchurch, New Zealand; Department of Wine, Food and Molecular Biosciences, Lincoln University, Christchurch, New Zealand

**Keywords:** Elaeis guineensis, Chromoplast, Fatty acid biosynthesis, Protein extraction, Gel electrophoresis, Proteomics

## Abstract

Proteins from the plant chromoplast are essential for many physiological processes such as fatty acid biosynthesis. Different protein extraction methods were tested to find the most robust method to obtain oil palm chromoplast proteins for mass spectrometry analysis. Initially, two different solvents were employed to reduce the fruit lipids. Then, two plant cell wall digestive enzymes were used to acquire the protoplasts to increase the protein extraction effectiveness. A two-stage centrifugation-based fractionation approach enhanced the number of identified proteins, particularly the fatty acid biosynthetic enzymes. The effectiveness of each extraction method was assessed using protein yields and 2DE gel profiles. The ideal method was successfully used to establish the 2DE chromoplast proteome maps of low and high oleic acid mesocarps of oil palm. Further nanoLC–MS/MS analysis of the extracted chromoplast proteins led to the identification of 162 proteins, including some of the main enzymes involved in the fatty acid biosynthesis. The established procedures would provide a solid foundation for further functional studies, including fatty acid biosynthetic expression profiling and evaluation of regulatory function.

**Electronic supplementary material:**

The online version of this article (doi:10.1186/s40064-015-1576-4) contains supplementary material, which is available to authorized users.

## Background

Oil palm (*Elaeis guineensis* Jacques) is the most important plant commodity in Malaysia, covering more than 5 million hectares in 2012. This oil crop is currently the world’s top commodity oil-bearing crop, with about 3.5 tons of oil per hectare produced annually in Malaysian plantations (Basiron 2012; Kirkland 2011; Barcelos et al. 2015). Palm oil is versatile and nutritious, free of *trans*-isomer fat and rich in vitamins and antioxidants (Hayes and Pronczuk 2010; Obahiagbon 2012; Sen et al. 2010). The export of palm oil products makes up between 9.3 and 11.5 % of Malaysia’s total exports and nearly 24.1 % of the global oils and fats export trade in 2011 (Palmoilworld 2014). Therefore, strategically designed and exhaustive research and development has been carried out to ensure the oil palm industry stays competitive and sustainable. On average, crude palm oil contains about 49 % of saturated fatty acids (palmitic acid, 44 % and stearic acid, 5 %) (Sundram 2000; Sundram et al. 2003; Sambanthamurthi et al. 2000). Reducing the saturated fatty acids by increasing the unsaturated fatty acid content, specifically oleic acid would add nutritional value to palm oil (Asemota et al. 2004; Dussert et al. 2013).

Chromoplasts develop from chloroplasts in ripened fruits (Bouvier et al. 1998), or directly from proplastids in other tissues (Ljubesic 1972). As the name suggests, they contain colored pigments (red, yellow and orange carotenoids) that produce an array of different colors to attract biotic vectors such as fruit-eating animals (Juneau et al. 2002). Plant fatty acid biosynthesis also occurs in the plastids, in both photosynthetic plastids such as leaf chloroplasts and in the non-photosynthetic plastids of flowers and fruits (Stumpf 1969; Joyard et al. 2010; Benning 2008, 2009). Recently, several proteomic investigations on fruit chromoplasts have been reported (Barsan et al. 2010; Hansen and Chiu 2005; Siddique et al. 2006; Tetlow et al. 2003).

Proteomic approaches are an effective way to study the protein composition and cellular functions of subcellular organelles such as chromoplasts. These techniques can be used to create protein maps at specific time point to give qualitative and quantitative profiles of the proteome. However, plant materials pose unique problems when it comes to protein extraction, hindering thorough proteomic analysis, which requires complete recovery of sample proteins. The low ratio of protein to cell mass [due to the presence of large vacuoles (Saravanan and Rose 2004) and interfering compounds such as lipids, polysaccharides and phenolics], presents an obstacle to protein extraction, possibly explaining the lack of studies on fatty acid biosynthesis regulation in high lipid-containing fruits, such as the oil palm *E. guineensis* var. Tenera examined in this study (Dussert et al. 2013).

Lipid-containing plant tissues require a labor intensive workflow to produce lipid-free proteins for proteomic studies (Wang et al. 2003, 2004, 2006). This is to ensure that the final protein preparation is free of interfering compounds to gel electrophoresis such as lipids. To maximize the protein yield from plant organelles, several research groups have used digestive enzymes to obtain “naked” protoplasts, which are essentially plant cells without the cell wall (Jain et al. 2008; Echeverria et al. 1985; Van der Wilden et al. 1980; Nishimura and Beevers 1978; Davey et al. 2005; Faraco et al. 2011). Such gentle cell wall disruption (compared to mechanical disruption) enables internal organelles to be maintained intact. This is crucial to ensure that only proteins from the chromoplast are extracted. The most widely applied contemporary method for protein extraction from recalcitrant plant tissues requires phenol extraction followed by protein precipitation with either ammonium acetate/methanol or trichloroacetic acid (TCA)/acetone. Proteins acquired in this way are suitable for proteomic analysis (Wang et al. 2003; Xie et al. 2007; Fan et al. 2009; Gomez-Vidal et al. 2009; He and Wang 2008). Identification of the chromoplast proteins is essential to enable functional annotation of the proteins based on their gene ontologies (GO) (Camon et al. 2003; Dutkowski et al. 2013; Harris et al. 2004; Consortium TGO 2008). Each identified proteins has a gene identifier assigned which can be used to retrieve their GO terms (biological process, molecular function and cellular components).

The purpose of the current work was to evaluate and modify existing methodologies to suit oil palm organelle proteomics, focusing on identifying the fatty acid biosynthetic enzymes. The methodologies developed may be able to facilitate comprehensive understanding of the regulation of fatty acid biosynthesis, and have provided a snapshot of the entire proteome of the oil palm fruit chromoplast.

## Results and discussion

### Mesocarp preparation

**Fig. 1:**
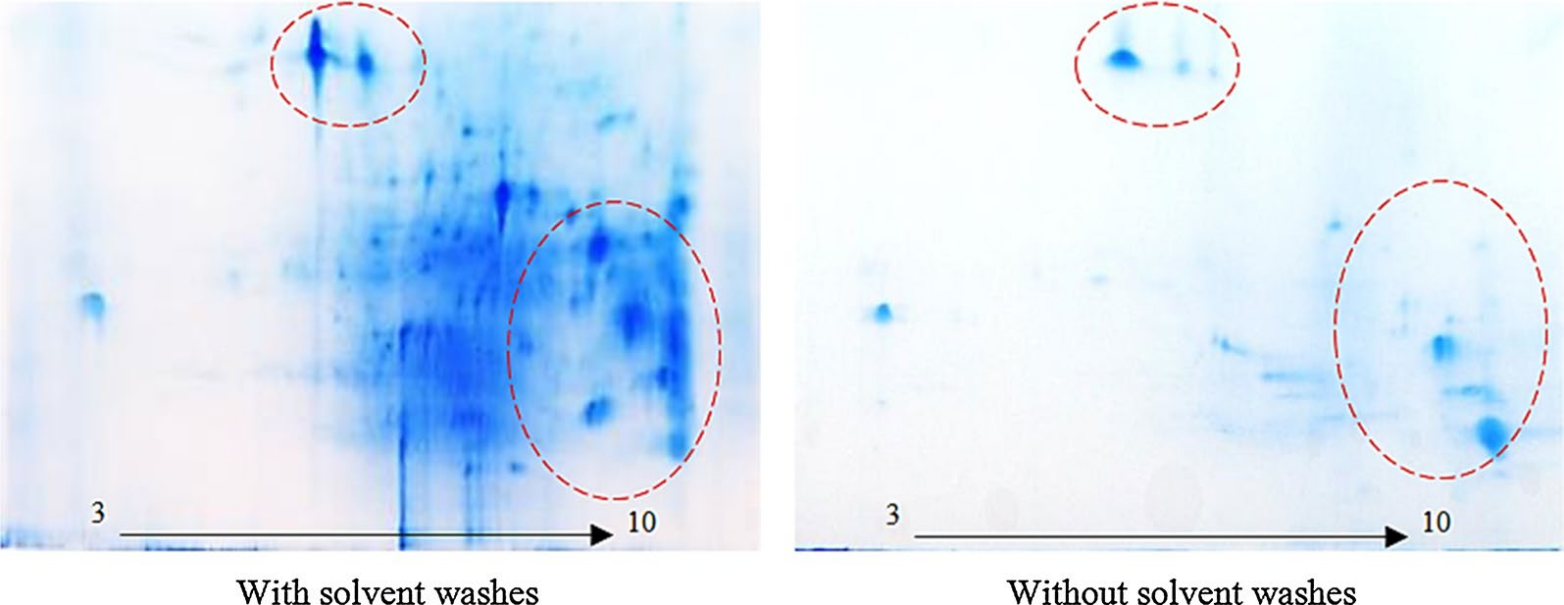
Effects of solvent washes on 2DE protein profiles from homogenized mesocarp tissues. Missing or different intensity protein spots are highlighted in *dotted circles*

To prepare high quality proteins from *E. guineensis* var. Tenera, a modification of the methods of Wang and co-workers (Wang et al. 2003, 2006; Gorg et al. 1997) was developed to suit oil palm mesocarp tissues. Finely ground mesocarp powder was delipidated using three washes with organic solvents (which were selected based on their capacity to extract different classes of lipids). The protein content was then measured by colorimetric assay. Protein yield from delipidated mesocarps was 1.16 ± 0.03 µg/µL compared to 0.71 ± 0.07 µg/µL obtained from un-delipidated mesocarps. This 1.6-fold increase in protein yield indicated that excess lipid greatly reduced the effectiveness of the protein extraction. 2DE gel profiles of mesocarp proteins with and without solvent washes (Fig. 1) clearly show that more proteins were extracted from delipidated mesocarps (based on the number and intensity of detected protein spots).

A combination of TCA and acetone was used in the first wash as this combination had been reported to be more effective than either TCA or acetone alone (Agrawal et al. 2010). Acetone dissolves simple lipids and glycolipids (Rastegari et al. 2011). This washing stage was limited to two washes only as prolonged exposure to low pH caused by TCA could potentially modify and/or degrade proteins. In the second wash, aqueous methanol in the presence of ammonium acetate salt was employed to remove more lipids from mesocarps. Polar solvents, such as methanol, are able to solubilize polar lipids like phospholipids and glycosphingolipids (Christie 1993). Aqueous methanol is also routinely used in extraction of phenolic compounds (Wang et al. 2006), which is advantageous for recalcitrant tissues since these tissues contain high amounts of phenolics. A combination of salt and organic solvent was employed in this washing stage to eliminate any residual TCA, and also to produce an alkaline pH for subsequent protein extraction using phenol (Wang et al. 2006). The wash involved the utilization of aqueous acetone to remove any residual lipids.

### Incubation with cell wall digestive enzymes (CDE)

**Fig. 2:**
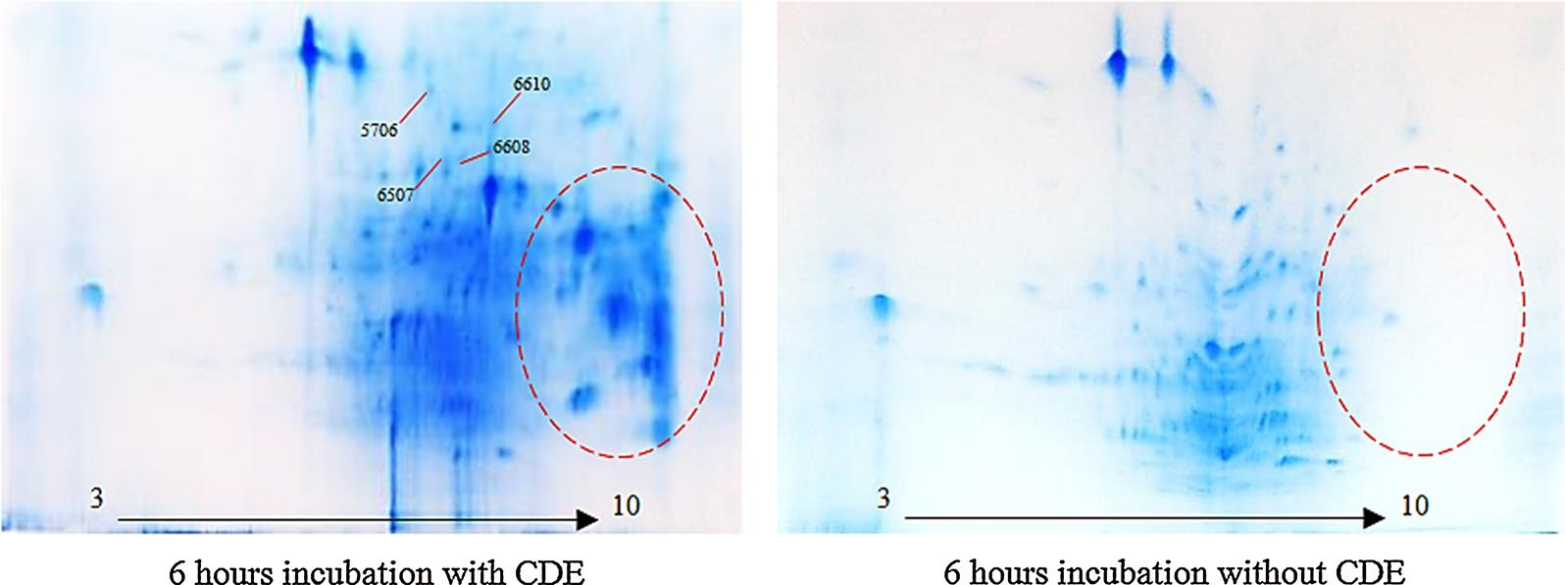
Effect of CDE in enhancing the number of protein spots in 2D gels. Missing protein spots in the basic region are highlighted in the *dotted circle*. Selected protein spots for comparative analysis using PDQuest are indicated

**Fig. 3:**
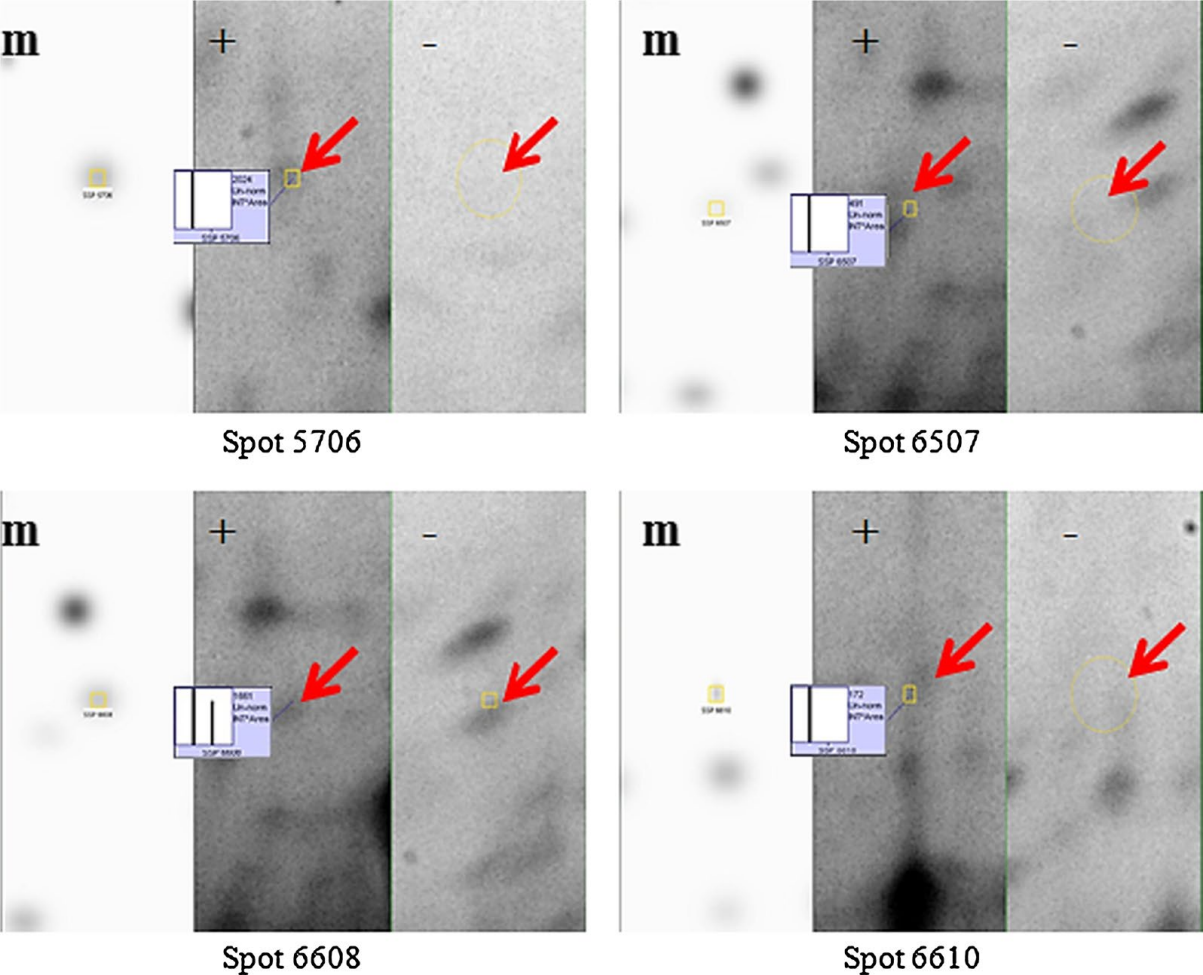
Comparative PDQuest protein spot analyses demonstrating differences in spot intensities of mesocarp proteins extracted with and without CDE digestion. From *left* Master gel (m); 6 h with CDE(+); 6 h without CDE(−). Protein spots are indicated with *red arrow* while their relative quantities are compared using the histograms

A combination of two cell wall digestive enzymes, cellulase and pectinase, was used to obtain protoplasts for subsequent chromoplast isolation. This clearly improved the protein yields from the solvent washed mesocarps. Protein yield was enhanced by approximately twofold from 0.64 ± 0.02 to 1.16 ± 0.02 µg/µL. The 2DE profiles and selected protein spot-to-spot comparisons of mesocarp proteins extracted with and without CDE after solvent washes supported these results (Figs. 2, 3).

**Fig. 4:**
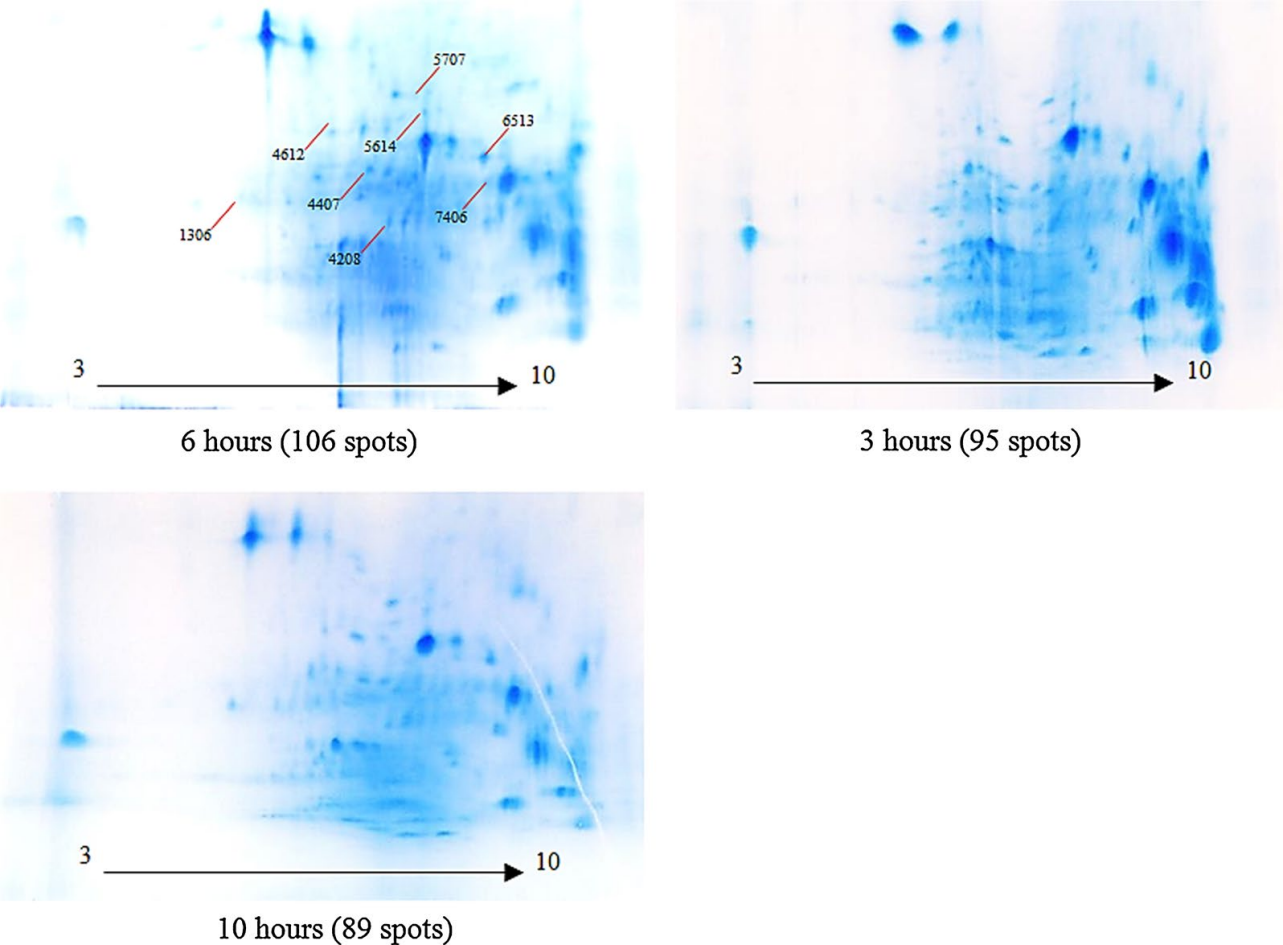
Outcomes of different CDE incubation periods of solvent-washed mesocarp tissues. Selected 2DE protein spots used for comparative analysis using PDQuest are indicated

We examined the effect of three different CDE incubation times. The protein yield of solvent-washed mesocarps incubated with CDE was 1.01 ± 0.03 µg/µL after 3 h, 1.16 ± 0.02 µg/µL after 6 h and 1.04 ± 0.01 µg/µL after 10 h. The extracted proteins were separated using 2DE (Fig. 4). The largest number of protein gel spots was observed after 6 h of CDE incubation (106 protein spots), compared to only 3 h (95 protein spots) and 10 h (89 protein spots).

**Fig. 5:**
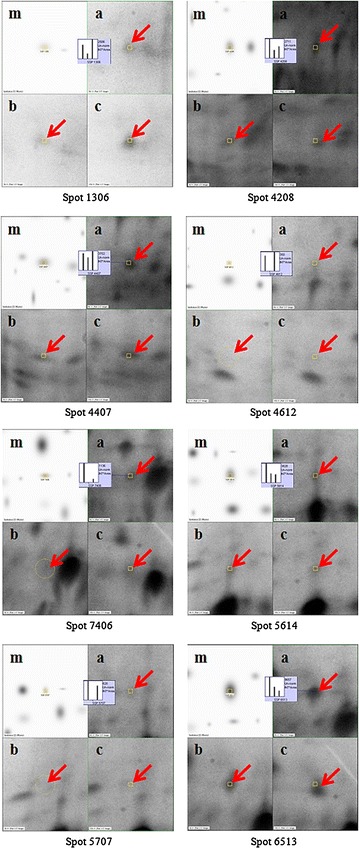
Comparative protein spot analysis with PDQuest demonstrating the effect of CDE incubation time on spot intensities. Clockwise from *left* Master gel (m); 6 h (*a*); 3 h (*b*); 10 h (*c*). Protein spots are indicated with red arrow while their relative quantities are compared using the histograms

Protoplast isolation (used to study plant organelles from several species (Van der Wilden et al. 1980; Agrawal et al. 2010; Chatterjee et al. 2012) may also be accomplished using mechanical disruption. Mechanical force protoplast isolation, however, is disadvantaged by high levels of contamination from protein storage vacuoles and mitochondrial proteins (Jain et al. 2008). For our application, we found gentler enzyme-driven cell wall disruption to be a suitable and effective approach. The enzymes used break down cellulose and pectin, the main components of the cell wall (Rastegari et al. 2011; Agrawal et al. 2010; Keegstra 2010). Long incubation times at 37 °C caused unspecific degradation of temperature-sensitive proteins, which may explain the lower protein yields observed after 10 h compared to 6 h. Limitation in the amount of mesocarp used in the study would resulted in CDE activity reaching a plateau after more than 6 h incubation and thus, prevent the yield from increasing further. A comparison of selected protein spot intensities in each set of the three gels (Fig. 5) also highlighted 6 h as the most suitable mesocarp/CDE incubation period to produce protoplasts.

### Organelle isolation

Following cell wall digestion, the predominantly chromoplast protein pellet was first obtained using differential centrifugation before further isolation with sucrose density gradient centrifugation (Barsan et al. 2010; Agrawal et al. 2010). The three clear interphases were retrieved and extracted proteins from each of the interphase were analyzed mass spectrometrically to assess the effectiveness of the strategy. As anticipated, the total number of identified proteins (25) and their peptides (78) increased almost fourfold to 93 proteins and 298 peptides using the two-stage isolation approach. Most importantly, nine fatty acid biosynthetic enzymes were identified, compared to only five using differential centrifugation alone. Conventionally, sucrose density gradient is applied to purify targeted organelle extract from other contaminant organelles co-extracted in the first isolation stage using differential centrifugation. However, in this study, the second isolation stage was intended to enhance the detection of fatty acid biosynthetic enzymes by fractionating the predominantly chromoplast suspension into three individual interphases. An overview of the identified proteins from whole mesocarp and chromoplast protein extracts showed that the combined interphases contained pre-dominantly chromoplast as it was not heavily cross-contaminated by other organelles. This was based on the non-detectable organelle protein markers (normally used as antibodies in Western blot analysis for degree of enrichment assessment) for mitochondria (cytochrome c oxidase) and endoplasmic reticulum (NADPH cytochrome c reductase) after the enrichment steps. However, cytosolic sucrose synthase one and peroxisome catalase two were identified, indicating possible cross-contamination by these organelles. Chloroplastic Rubisco large subunit were present in both whole mesocarp and chromoplast extracts (Additional file 1, Additional file 2). These markers were identified mass spectrometrically.

### Organelle protein extraction

Proteins were extracted both from the chromoplast pellet and the interphases obtained by sucrose density gradient centrifugation. Proteins from the chromoplast pellet were also used to evaluate the effectiveness of several solutions for protein extraction. 100 % phenol, phenol with sodium dodecyl sulfate (phenol/SDS), SDS alone, and TCA/acetone were used to extract the proteins (Rastegari et al. 2011; Rodrigues et al. 2012; Méchin et al. 2006; Yeung et al. 2008). Precipitation of the proteins after extraction was performed with either ammonium acetate/methanol or TCA/acetone.

Table 1 shows the protein yields generated from the various combinations of protein extraction and precipitation approaches. The yield from SDS alone and TCA/acetone extractions could not be determined, since the protein pellets could not be dissolved in non-urea-containing buffer. Phenol/SDS extraction followed by precipitation with ammonium acetate/methanol outperformed 100 % phenol extraction in terms of protein yield. Nevertheless, 100 % phenol extraction was selected for further method development, due to the risk of SDS interfering with isoelectric focusing and mass spectrometric analysis (Yu et al. 2003; Carpentier et al. 2005). Ammonium acetate/methanol precipitation was selected for progression because TCA/acetone-precipitated protein pellets were difficult to resolubilize (Rastegari et al. 2011).

**Table 1:** Protein yields using different protein extraction approaches

Extraction	Precipitation	Protein yield (µg/µL)
100 % phenol	Ammonium acetate/methanol	1.84 ± 0.09
Phenol/SDS	Ammonium acetate/methanol	2.22 ± 0.05
SDS alone	Ammonium acetate/methanol	Not determined
100 % phenol	TCA/acetone	2.10 ± 0.03
TCA/acetone	None	Not determined

Non-phenol extracts formed yellow to brownish precipitates due to polyphenol oxidation (Saravanan and Rose 2004; Wang et al. 2003; Carpentier et al. 2005). Precipitates from phenol protein extractions were whitish in color, indicating the absence of polyphenols. Plant metabolites such as phenolics are capable of forming hydrogen bonds and irreversible complexes with proteins through oxidation and covalent condensation (Loomis and Battaile 1966). TCA/acetone is a highly effective protein precipitation method (Rastegari et al. 2011) but this approach is not suitable for the elimination of polyphenols and lipids from plants. Several studies also mentioned the unsuitability of TCA/acetone in extracting proteins from complex tissues due to polyphenolic oxidation for instance (Saravanan and Rose 2004; Wang et al. 2003; Carpentier et al. 2005).

**Fig. 6:**
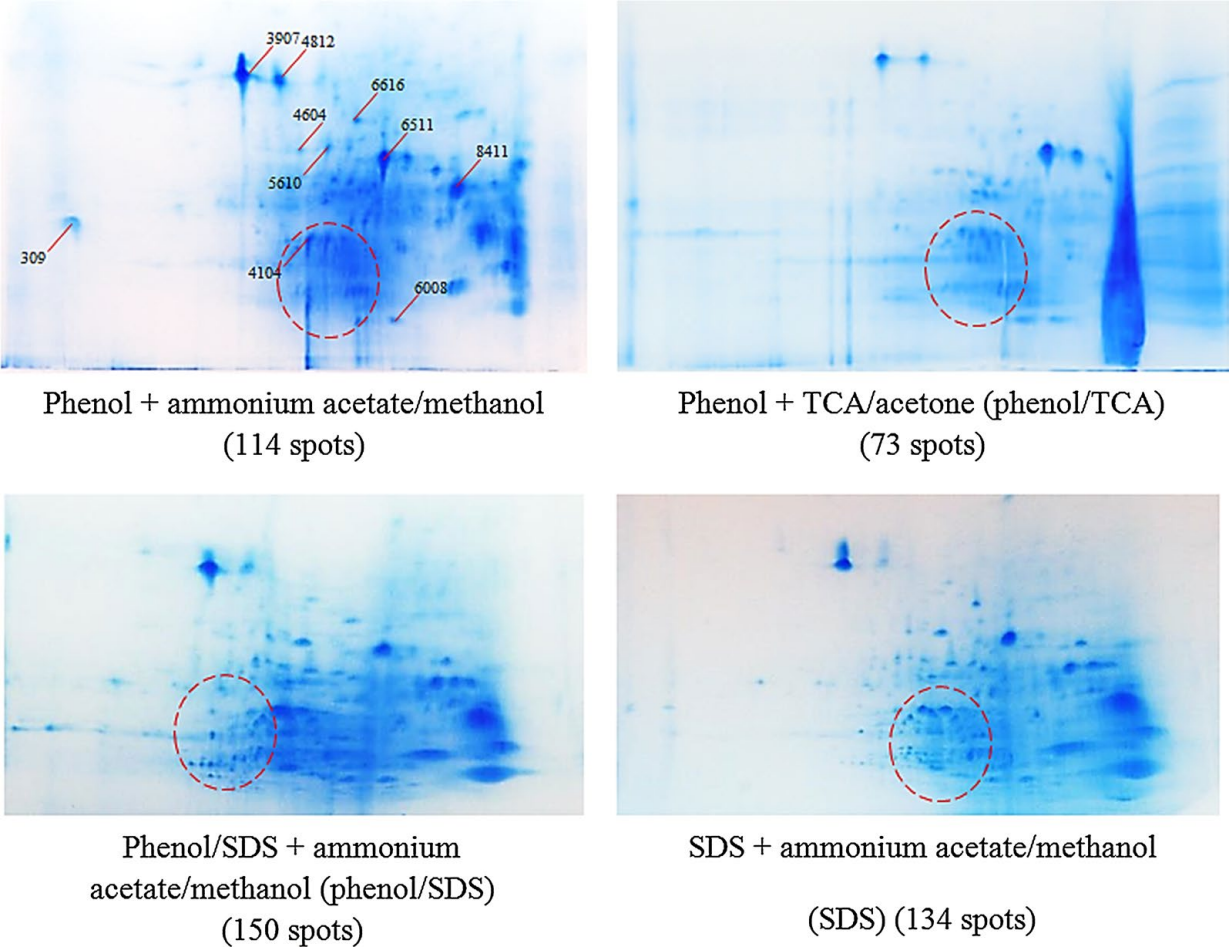
Effect of different extraction approaches on 2DE protein profiles. PDQuest comparative analysis was performed on selected protein spots. Missing or different intensity spots are highlighted in *dotted circles*

**Fig. 7:**
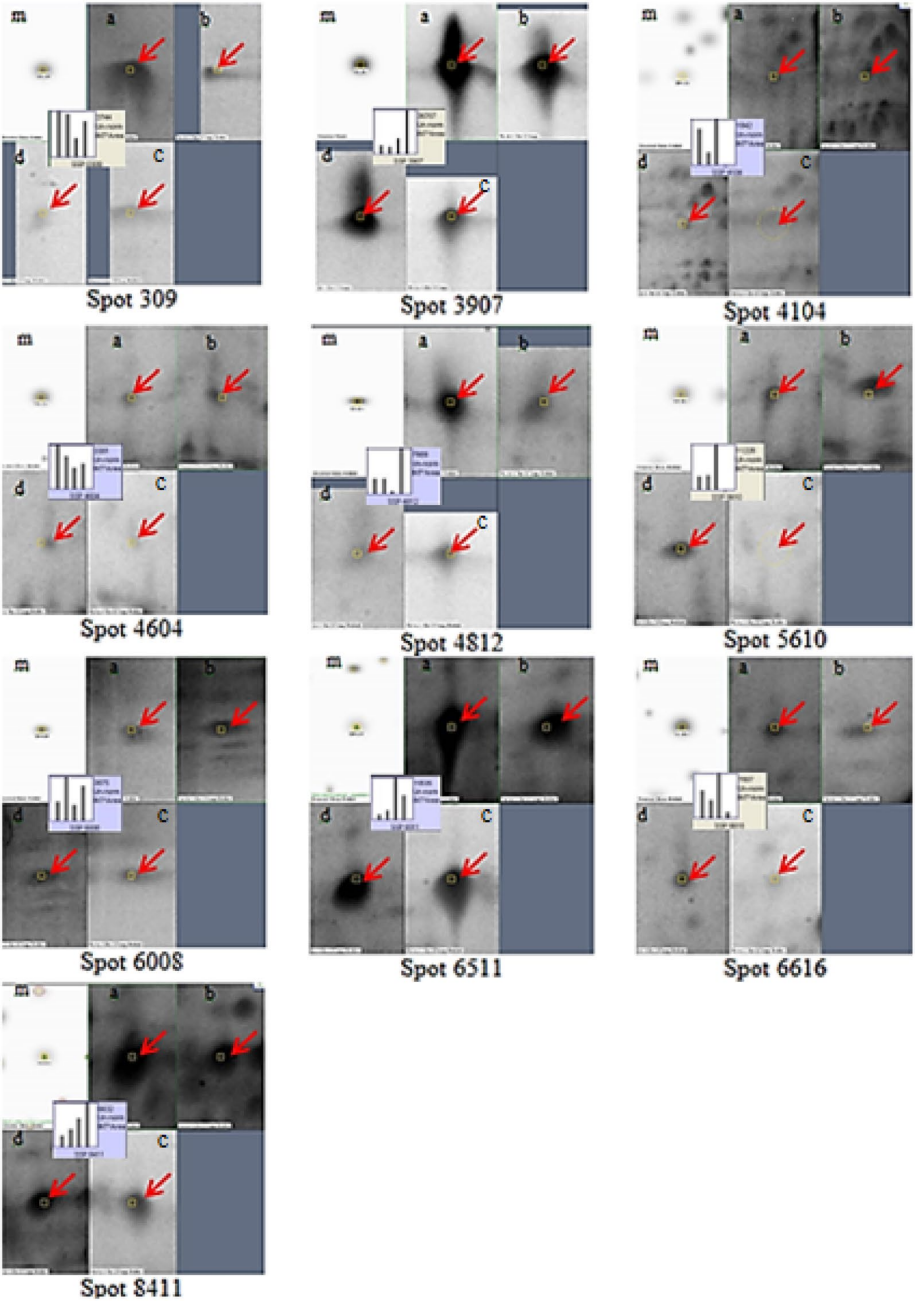
Comparative PDQuest spots analysis demonstrating several protein spots that exhibit intensity differences from the various extracts. Clockwise from *left* Master gel (m); 100 % phenol (*a*); phenol/SDS (*b*); SDS alone (*c*); phenol/TCA (*d*). Protein spots are indicated with *red arrow*

The protein extracts were also evaluated by comparing their 2DE protein profiles. The number of 2D gel protein spots correlated well with the protein yields for each of the extraction methods. With ammonium acetate/methanol precipitation, extraction with 100 % phenol generated 114 protein spots compared to phenol/SDS (150 protein spots) and SDS alone (134 protein spots) (Fig. 6). 100 % phenol extraction combined with TCA/acetone precipitation produced only 73 protein spots. TCA/acetone-extracted proteins were not suitable for 2DE analysis since the precipitate could not be dissolved even in urea-containing buffer. These results indicate that SDS enhanced protein extraction, but this was countered by poor protein spot resolution in the phenol/SDS and SDS alone extracts (SDS is known to be detrimental to isoelectric focusing (Kitajima and Sato 1999; Liu and Ekramoddoullah 2006). Gel spots from 100 % phenol extracts had better resolution and less streaking, especially in the basic region. Since the fatty biosynthetic enzymes that we are interested in are mostly basic proteins, we opted to forgo the advantage offered by SDS in term of protein yields and to use 100 % phenol extraction to maximize the electrophoretic resolution of basic proteins. The selection of the best precipitation method following protein extraction was based on their 2D gel performance. 100 % phenol extraction followed by TCA/acetone precipitation yielded low numbers of protein spots and poor resolution, along with protein aggregation and severe vertical streaking in the basic region of the 2D gel. Ammonium acetate/methanol precipitation performed much better in this regard. Spot-to-spot comparisons (PDQuest) were performed for selected protein spots (Fig. 7). Protein spot intensities were similar in the 100 % phenol, phenol/SDS and SDS alone extractions followed by ammonium acetate/methanol precipitation. However, the protein intensities of the same protein spots for TCA/acetone precipitated proteins after 100 % phenol extraction were lower. A summary of the advantages and disadvantages of each approach tested is presented in Table 2.

**Table 2:** Summary of different protein extraction and precipitation approaches

Extraction	Precipitation	Protein yield	2DE gel spots	Pellet solubility	Mass spectrometry interference
100 % phenol	Ammonium acetate/methanol	Good	114	Good	No
100 % phenol	TCA/acetone	Good	73	Difficult	No
Phenol/SDS	Ammonium acetate/methanol	Best	150	Good	Possible
SDS alone	Ammonium acetate/methanol	Not determined	134	Poor	Possible
TCA/acetone	–	Not determined	Not suitable for 2DE	Poor	No

### Biological processes of oil palm chromoplast proteins

**Fig. 8:**
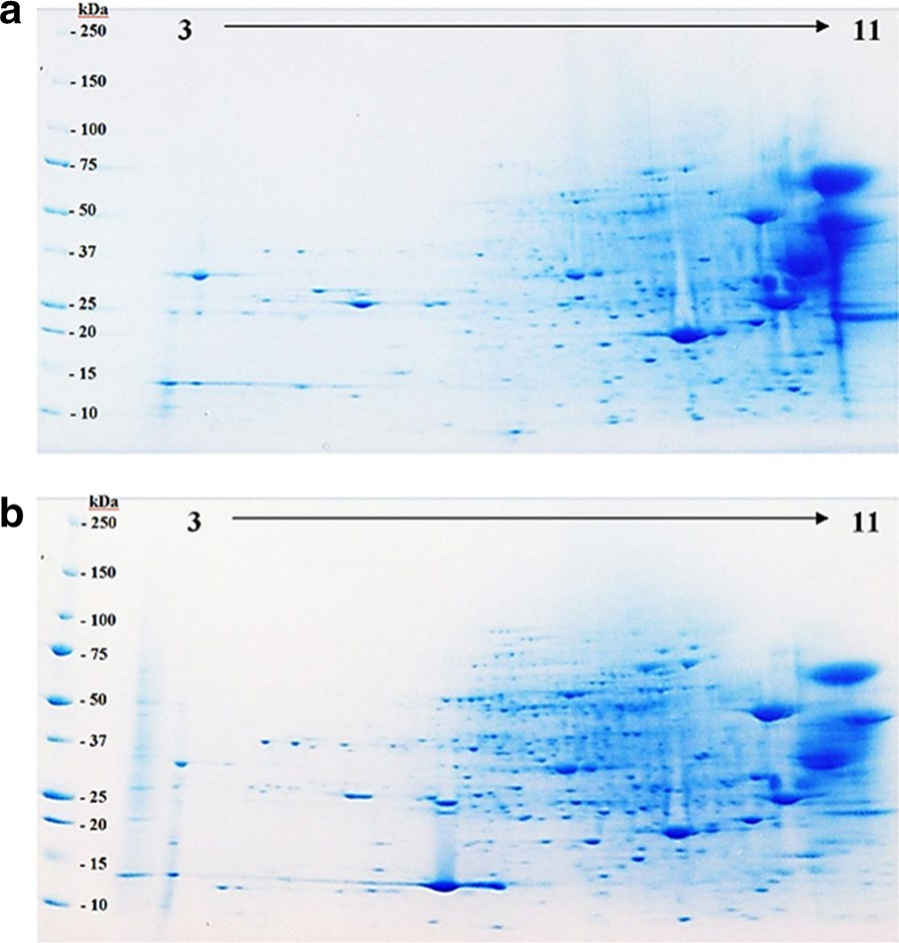
2DE proteome maps of the mesocarp chromoplast. **a** Low oleic acid mesocarp and **b** high oleic acid mesocarp. pH range (3–11) and the molecular marker weights are indicated

**Fig. 9:**
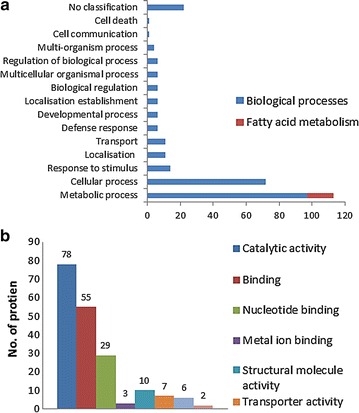
**a** Biological processes and **b** molecular functions associated with chromoplast proteins

The customized chromoplast isolation and protein extraction methodology was applied to generate the first 2D proteome maps for low and high oleic mesocarps (Fig. 8). The proteome maps were crucial in assessing the protein profiles for these two fruit mesocarps to detect differentially expressed proteins due to different level of oleic acid. Identified proteins from both low and high oleic acid mesocarps using gel-free LC-MS/MS approach were compiled to yield 162 non-redundant proteins (Additional file 2). These proteins were further categorized according to their biological process (Fig. 9a) and molecular functions (Fig. 9b) by mapping the respective peptide sequence gene identifier number to existing annotations of characterized proteins (Camon et al. 2003; Dutkowski et al. 2013; Harris et al. 2004). The proteins reported here represent just a portion of the entire chromoplast proteome since there are still many lower abundant proteins yet to be discovered. The high proportion of proteins assigned to metabolic (97 proteins) and cellular processes (72 proteins) corresponds well to the high number of proteins (78) with catalytic activity. Sixteen of these proteins are understood to be involved in fatty acid metabolism. Main fatty acid biosynthetic enzymes identified were acetyl-CoA carboxylase, 3-enoyl-ACP reductase, 3-hydroxyacyl-ACP dehydrogenase, β-ketoacyl-ACP reductase and stearoyl-ACP desaturase. Our results indicated that at least two fatty acid biosynthetic enzymes are unique to either low or high oleic acid mesocarp. Fifty-five of the proteins had binding activity, which explains the presence of numerous pathogenesis-related proteins in the chromoplasts (Kitajima and Sato 1999; Liu and Ekramoddoullah 2006). Twenty-two proteins were not categorized and were denoted as “no classification”. These proteins were mostly homologs of unnamed or hypothetical proteins from *Zea mays*.

## Conclusions

We present here a detailed methodology optimized to prepare oil palm chromoplasts for 2DE analysis and mass spectrometry. Oil palm fruit mesocarps are recalcitrant tissues for proteomic analysis. Their high lipid content makes protein extraction challenging, and the plant material contains high levels of interfering compounds such as polysaccharides and phenolics. The method development described here was tailored specifically for *Elaeis guineensis* var. Tenera fruit mesocarps to enhance overall protein yield and fatty acid biosynthesis-associated enzymes for downstream proteomic characterization. Removal of lipids from the mesocarp proved to be critical in increasing the effectiveness of protein extraction. Also the incorporation of two cell wall digestive enzymes (CDE) helped reduce the difficulty posed by plant cell wall in efficient protein extraction. Subsequently, the use of 100 % phenol in extracting the chromoplast proteins, followed by protein precipitation with ammonium acetate in methanol was revealed to be the most suitable approach to acquire mass spectrometry-compatible proteins. Chromoplast proteome maps from two oil palm cultivars, namely, the low and the high oleic acid mesocarps, are presented. In our search for the fatty acid biosynthetic enzymes, we introduced two centrifugation-based isolation stages and compiled identified proteins with 95 % confidence level from the two oil palm fruit mesocarps to identify 16 major fatty acid biosynthetic enzymes for further analysis of their roles in regulation. Functional annotations of those proteins indicated that 9.8 % of the identified proteins were implicated in fatty acid biosynthesis. Loei and co-workers had reported protein extraction from oil palm fruit mesocarps for the protein expression profiling (Loei et al. 2013) but a detailed method development to extract proteins from oil palm fruit chromoplasts has never been described before.

## Methods

### Materials

Fruits of the commercial Dura x Pisifera oil palm crosses (*Elaeis guineensis* var. Tenera) (denoted as low oleic acid mesocarp) and from the MPOB Breeding Population 12 (denoted as high oleic acid mesocarp with 20 % more oleic acid content) were grown and harvested at the Malaysian Palm Oil Board research stations at Bangi, Selangor and Hulu Paka, Terengganu, Malaysia. Oil palm bunches of 20th week after anthesis were collected from three low and three high oleic acid mesocarps, and used as the biological replicates. The fruit mesocarps were sliced, snap-frozen in liquid nitrogen and stored at −80 °C until further use. For the purpose of method evaluations, only the low oleic acid mesocarp was used.

### Removal of lipid by different organic solvents

A lipid removal procedure from oil palm samples was modified from the method by Wang and co-workers (Wang et al. 2006) to suit mesocarp tissue. Five grams of sliced mesocarp (average size of 1 cm in diameter) were homogenized in liquid nitrogen with a cold Waring blender (Dynamics Corporation, Greenwich, USA) at low grinding speed for 10 s, followed by hand grinding with a ceramic mortar and pestle. The powdered mesocarp was mixed with cold acetone containing 10 % (w/v) trichloroacetic acid and 1 mM dithiothreitol. The slurry was then centrifuged (RA-300 rotor, Kubota 7820, Kubota Corporation, Tokyo, Japan) at 13,000*g* for 10 min at 4 °C. The supernatant was discarded and the washing step repeated once. Cold 80 % (v/v) methanol containing 0.1 M ammonium acetate was added to the precipitate, mixed and centrifuged as before. After the supernatant was discarded, the precipitated mesocarp pellet was washed with cold 80 % (v/v) acetone. The mixture was mixed well and centrifuged at 13,000*g* for 10 min at 4 °C. The final washed mesocarp pellet was air-dried.

### Isolation of chromoplasts

The isolation method of chromoplasts was adapted from Jain, Fan and co-workers (Jain et al. 2008; Fan et al. 2009). The washed mesocarp pellet was transferred into a beaker containing 15 mL of cell wall digestive enzymes (CDE) [2 % (w/v) cellulase (0.8 U/mg, Sigma-Aldrich, Co., MO, USA), 0.1 % (w/v) pectinase (1 U/mg, Sigma-Aldrich, Co., MO, USA), 0.6 M sorbitol, 0.1 M dithiothreitol, 5 mM 2-(4-morpholino)-ethane sulfonic acid-KOH, pH 5.5]. The suspension was then gently agitated for 6 h at 37 °C. After cell wall digestion, the mixture was sieved through two layers of Mirocloth (Calbiochem, EMB Millipore Corporation, MA, USA) into a beaker on ice to separate non-macerated plant materials from the protoplasts. The filtrate was centrifuged at 1750 g for 5 min at 4 °C to collect intact chromoplasts. The chromoplast pellet was gently re-suspended in the extraction buffer containing 0.7 M sucrose, 1 M Tris–HCl, pH 8.3, 5 M NaCl, 50 mM dithiothreitol, 1 mM EDTA and Roche protease inhibitor cocktails (1 tablet to 10 mL of buffer) (Roche Diagnostics GmbH, Mannheim, Germany). The mixture was agitated gently to avoid the disruption of the chromoplasts before the second stage of isolation based on the procedure by Barsan and co-workers (Barsan et al. 2010). The re-suspended pellet in extraction buffer was loaded onto a sucrose discontinuous gradient made from 1.2, 1.7 and 2.2 M sucrose. After that, they were centrifuged (JA-30.50 rotor, Avanti J-301, Beckman Coulter, CA, USA) at 62,000*g* for 45 min at 4 °C to yield three interphases containing predominantly chromoplasts. These interphases were carefully retrieved for protein extraction.

### Organelle protein extraction

The chromoplast pellet (from the first stage of isolation) and all the three interphases (from the second stage of isolation) were used for subsequent proteomic analyses. The extraction protocol remained the same regardless of the protein sources. The re-suspended chromoplast pellets from the extraction buffer or the interphases were sonicated for 15 min at 4 °C to break the chromoplasts. An equal volume of fresh 50 mM Tris-saturated phenol, pH 8, was added to the mixture. The mixture was agitated for 10 min before centrifuging at 15,000*g* for 15 min at 4 °C to separate the phenol and Tris phases. Following centrifugation, the upper phenol phase was transferred to a new tube and the proteins precipitated by the addition of five volumes of cold 0.1 M ammonium acetate-saturated methanol, followed by incubation at −20 °C overnight. A protein pellet was obtained by centrifuging at 15,000*g* for 15 min at 4 °C. The pellet was rinsed until whitish with cold 0.1 M ammonium acetate-saturated methanol, and then washed three times with cold 80 % (v/v) acetone. Proteins were precipitated after each wash by centrifuging at 15,000*g* for 5 min at 4 °C. The chromoplast protein pellet was air-dried. For TCA/acetone protein precipitation, Acetone A [containing 10 % TCA (w/v) and 1 mM DTT (w/v)] was added to the protein solution. Acetone B [containing 1 mM DTT (w/v)] was used to wash the protein pellet prior to drying. A commercially available 2DE Quant kit (GE Healthcare Life Sciences, Uppsala, Sweden) was used for protein estimation (µg/µL). Bovine serum albumin provided in the kit was used as the protein calibration standard and three technical repeats for each quantitation were performed.

### Two-dimensional gel electrophoresis (2DE)

2DE was used to illustrate the effect of lipid removal and cell wall digestive enzymes (CDE), as well as the effect of CDE incubation period on protein extraction. The extraction approaches were evaluated in terms of the number of protein spots obtained and their intensities. Air-dried chromoplast protein pellets were solubilized in rehydration buffer containing 7 M urea, 2 M thiourea, 4 % (w/v) 3-[(3-cholamidopropyl)dimethylammonio]-1-propanesulfonate (CHAPS), 0.25 % (v/v) broad range pH 3-10 Pharmalyte and 0.4 % (w/v) dithiothreitol. Protein quantitation was performed prior to the first dimension of 2DE; isoelectric focusing (IEF).

*Gel maps for method development:* IEF was performed using Bio-Rad ReadyStrip™ IPG strips, 7 cm, pH 3-10 (Bio-Rad Laboratories Inc., CA, USA), which were passively rehydrated with 100 µg of protein in 125 µL of rehydration buffer overnight. IEF was performed using a Bio-Rad Protean IEF Cell (Bio-Rad Laboratories). A total of 10,000 volt-hours were used to focus the proteins. After IEF, the proteins were reduced in 1 % (w/v) dithiothreitol for 15 min followed by alkylation with 4 % (w/v) iodoacetamide for 15 min before SDS-PAGE. The second dimension of 2DE was carried out using in-house packed 1.0 mm 12 % polyacrylamide gels.

*Two*-*dimensional proteome maps of the low and high oleic acid oil palm mesocarps*: Bio-Rad ReadyStrip™ IPG strips, 11 cm, pH 3–11 were passively rehydrated with 400 µg of protein in 200 µL of rehydration buffer overnight. IEF was performed using a Bio-Rad Protean IEF Cell. A total of 20,000 volt-hours were used to focus the proteins. The focused proteins were reduced as described above. The IEF strips were laid on top of Precast Mini-PROTEAN^®^ TGX™, 1.0 mm 4–20 % polyacrylamide gradient gels (Bio-Rad Laboratories) to generate high resolution proteome maps.

Electrophoresis (for both the method development gels and the 2DE proteome maps) was conducted in a Bio-Rad Mini-PROTEAN^®^ Tetra Cell apparatus (Bio-Rad Laboratories) at 200 V (Powerpac 300, Bio-Rad Laboratories). The separated proteins were fixed in the gels for 30 min [50 (v/v) ethanol, 10 % (v/v) acetic acid] and stained with Colloidal Coomassie G-250 (Candiano et al. 2004). The gels were de-stained in water until the background was clear. Gel images were obtained with a DLSR camera (Nikon D100, Nikon Corporation, Tokyo, Japan). The settings were ISO 200, f16 for the aperture and a shutter speed of 1/40th of a second. Image processing was done with analySIS software (Soft Imaging System GmbH, Germany). Protein spot detection and subsequent protein spot-to-spot comparative analysis were done with PDQuest 2D analysis software (Bio-Rad Laboratories). The reproducibility of the 2DE protein profiles was determined using three independent biological replicates for low and high oleic acid oil palm mesocarps.

### In-solution protein digestion assisted by sodium deoxycholate

Prior to tryptic digestion, 50 µg of the chromoplast protein pellet or the interphase proteins in 0.1 M ammonium bicarbonate was reduced and alkylated with thiol-free 50 mM tris(2-carboxyethyl)phosphine and 55 mM iodoacetamide. An ionic detergent, 1 % (w/w) sodium deoxycholate (Koehn et al. 2011) was added to improve peptide solubilization. Proteins were digested with modified sequencing grade trypsin (Promega, WI, USA) for 16 h at 37 °C. After digestion, 0.5 % (v/v) formic acid was added to precipitate the sodium deoxycholate and then the digest was centrifuged at 14,000*g* for 15 min. The peptides were then dried in a centrifugal concentrator and kept at −80 °C until required.

### Liquid chromatography-tandem mass spectrometry (LC–MS/MS)

Peptide separation was performed with a nano-Advance liquid chromatography (LC) system (Bruker Daltonik GmbH, Bremen, Germany). The tryptic digests were re-suspended in 30 µL of 5 % (v/v) acetonitrile and 0.05 % (v/v) trifluoroacetic acid in 0.1 % (v/v) formic acid; 5 µL was loaded onto the precolumn. Peptide separation was performed with an in-house packed C18 Phenomenex Aeris XB trap column (3 µm, 0.1 × 100 mm) (Phenomenex Inc., CA, USA) and a prepacked Magic C18 AQ analytical column (3 µm, 0.1 × 150 mM) (Michrom Bioresources, Inc., CA, USA). Equilibration was carried out with 95 % solvent A (2 % acetonitrile, 0.1 % formic acid) and 5 % solvent B (98 % acetonitrile, 0.1 % formic acid). A 0–45 % solvent B gradient over 45 min at a flow rate of 800 nL min^−1^ was employed to elute the bound peptides, which were analyzed using an amaZon speed ion trap mass spectrometer (Bruker Daltonik). The capillary spray voltage used was 1300 V at a temperature of 150 °C. Mass spectrometric survey scans were performed to acquire precursor ions with a mass range from *m*/*z* 310–1400. The resolution was set to “Enhanced Resolution” with a scanning speed of *m*/*z* 8100 s^−1^. Tandem MS spectra acquisition conditions consisted of “Xtreme Resolution” scan with a scanning speed of *m*/*z* 52,000 s^−1^ and a mass range of *m/z* 100–3000 with up to three of the most intense multiple charged precursor ions (1 + 2 + and 3 +) per scan fragmented (collision induced) in the linear ion trap. All MS/MS spectra were collected using a fragmentation amplitude of 1.0 V and an isolation window width of *m*/*z* 4.0.

### Protein identification and data analysis

Data acquisitions (in positive ion mode) were performed using Compass 1.3 for Amazon trapControl Version 7.0 (Bruker Daltonik). Data analysis to annotate and generate peak lists was performed using amaZon Data Analysis software Version 4.0 (Bruker Daltonik). The peak lists were then sent to ProteinScape Version 3.1 (Bruker Daltonik) for protein identification using Mascot Version 2.4.0 server (Matrix Science, Boston, MA, USA) (http://www.matrixscience.com). The peptide sequences were searched against Magnoliophyta taxonomy in the NCBInr protein database (http://www.ncbi.nlm.nih.gov). Error mass tolerances for protein and peptide were set to 0.3 and 0.6 Da, respectively. Semi-trypsin was designated as the cleavage enzyme with two missed cleavages allowed. Carbamidomethylation of cysteine (C) was set as the fixed modification while oxidation of methionine (M) and deamidation of asparagine (N) and glutamine (Q) were used as variable modifications. Protein identities were automatically accepted if they had at least one top ranking peptide (Rank 1) with an ion score identity threshold of more than 50 (*p* < 0.05), indicating correct protein matches. All database searches were performed against the decoy database (randomized sequences) as well for any false positive hits (to yield a FDR of <2 %). Only those proteins meeting the above protein assessment criteria were used for downstream analyses. The non-redundant proteins list was compiled by Protein Extractor module in ProteinScape bioinformatics platform.

### Functional classes assignment

Proteins were classified into their biological processes and molecular functions based on their gene ontology (GO) term. Their respective peptide sequence gene identifier numbers from the UniProtKB and NCBInr protein databases were mapped to the existing annotations of characterized proteins (Harris et al. 2004; Camon et al. 2003, 2004) using the gene ontology functionality provided with the ProteinScape platform.

## Additional files

10.1186/s40064-015-1576-4 Total proteins from whole oil palm fruit mesocarps.
10.1186/s40064-015-1576-4 Total protein from oil palm fruit mesocarps after two-step centrifugations.

## Abbreviations

2DE: two-dimensional gel electrophoresis
TCA: trichloroacetic acid
CDE: cell wall digestive enzyme
SDS: sodium dodecyl sulphate
